# Magnetic Excitation for Coupled Pendulum and Piezoelectric Wave Energy Harvester

**DOI:** 10.3390/mi16030252

**Published:** 2025-02-24

**Authors:** Wuwei Feng, Xiang Luo, Shujie Yang, Qingping Zou

**Affiliations:** 1 College of Marine Engineering Equipment, Zhejiang Ocean University, Zhoushan 316004, China; fengwuwei@163.com (W.F.); luoxiang@zjou.edu.cn (X.L.); shujie.yang@zjou.edu.cn (S.Y.); 2 The Lyell Centre for Earth and Marine Science and Technology, Institute for Infrastructure and Environment, Heriot-Watt University, Edinburgh EH14 4AS, UK

**Keywords:** wave energy, piezoelectric energy harvesting, magnetic excitation, spring pendulum, PWEC, piezoelectric wave energy converter

## Abstract

Wave energy is one of the most reliable and promising renewable energy sources that has attracted lots of attention, including piezoelectric wave energy harvesting devices. One of the challenges for piezoelectric wave power generation is the relatively low-frequency wave environments in the ocean. Magnetic excitations are one of the techniques used to overcome this issue. However, there is a lack of understanding of the mechanisms to maximize the electric power output of piezoelectric wave energy harvesters through magnetic excitations. In the present study, magnetic excitation experiments were conducted to investigate the power generation of a coupled spring pendulum piezoelectric energy harvester under various magnetic field conditions. Firstly, the mass of the load magnet that can induce the resonance phenomenon in piezoelectric elements was experimentally determined. Then, the power generation of piezoelectric elements was tested under different excitation magnetic spacings. Finally, the influence of different distribution patterns of excitation magnets on the performance of piezoelectric elements was tested. It was found that under the conditions of a load magnet mass of 2 g, excitation magnet spacing of 4 mm, and two excitation magnets stacked on the inner pendulum, optimum power generation of the piezoelectric wave harvester was achieved with a peak-to-peak output voltage of 39 V. The outcome of this study provides new insight for magnetic excitation devices for piezoelectric wave energy harvesting to increase the feasibility and efficiency of wave energy conversion to electrical energy.

## 1. Introduction

In the past decades, the energy crisis and greenhouse effects have become pressing social and environmental issues. Wind, solar, tide, and wave energy are now popular environmentally friendly alternative energy resources [1]. Ocean wave energy, in particular, has great potential for development due to its high content in the ocean and predictable nature [2,3,4,5,6]. Marine countries invest heavily in wave energy generation technology. However, the commercialization of wave energy is hindered by its relatively low power generation efficiency, high manufacturing and maintenance costs, and poor reliability and stability. The low conversion efficiency of energy conversion at all levels in the renewable devices urgently needs to be resolved [7].

In the past two decades, micropower generation technology has made noticeable progress in the fields of microelectromechanical systems, self-powered wireless sensor nodes, and mobile platforms [8]. Batteries are the main power source for these systems, but have limitations, such as short lifespan, frequent replacement, and environmental pollution [9,10]. Therefore, battery systems are not suitable for the wireless self-powered electronics. Renewable energy, such as using mechanical vibrations to harvest energy, is an attractive alternative solution to extend the lifespan or replace batteries [11,12]. Extensive effort has been dedicated to vibration-based micropower generation. Electromagnetic, frictional, and piezoelectric transducers have been used to convert mechanical energy into electrical energy.

The electromagnetic generator was created based on an electromagnetic induction mechanism, and its working principle mainly involves changing the magnetic flux to an electromotive force to generate electrical energy. Kurt et al. [13] proposed a broadband electromagnetic energy harvester with a power generation of 17 μW at a vibration frequency of 60 Hz. Ordonez et al. [14] proposed a high-performance electromagnetic vibration energy harvester based on a ring magnet, using a Halbach array with a power generation of up to 61.7 mW. Although the electromagnetic generator produces high power, it requires a high vibration frequency, and its structure is relatively complex. It is difficult to achieve high power at low frequencies and balance power and frequency.

Friction generators generate electrical energy through the charging pump effect of frictional point potential. When two materials come into contact with each other, the charge separation between the two layers of polymer forms a potential difference, which in turn generates electricity. Demircioglu et al. [15] proposed a frictional electric nanogenerator for simulating energy harvesting under wave conditions. He used a pendulum-shaped device to collect wave energy and obtained a maximum voltage of 145 V and a maximum current of 8.9 μA. Compared to electromagnetic power generation, frictional electricity has the advantages of small size and better conversion of mechanical energy at low frequencies into electrical energy. However, frictional electricity device performance can be greatly affected by factors such as materials, humidity, and temperature, as well as low effective output power due to the high output voltage but low current, and the relatively complex structure.

Piezoelectric power generation relies on the positive piezoelectric effect of piezoelectric materials to convert mechanical energy into electrical energy. The positive piezoelectric effect is a phenomenon in which a crystal polarizes internally in a fixed direction under the action of external mechanical forces. As a result, a piezoelectric material generates positive and negative charges at its two surfaces, forming a potential difference. When the mechanical force is removed, the piezoelectric material returns to an uncharged state. Chen et al. [16] proposed a magnetic chaotic pendulum structure to enhance the absorption of external vibration frequencies through magnets, resulting in a power generation of 1.25 mW. Shi et al. [17] proposed a floating piezoelectric electromagnetic hybrid wave vibration energy harvester driven by a swinging ball. The frequency of the piezoelectric coupled cantilever beam was amplified by magnetic excitation, and then higher voltage at lower frequencies of waves was achieved by connecting it in series with a copper coil. Liu et al. [18] developed a low-frequency non-contact rotating piezoelectric generator with magnetic coupling excitation. By driving the coupling between the magnet inside the rotor and the magnet on the piezoelectric plate through rotational motion, the mechanical energy of the rotational motion was converted into electrical energy with a power generation of 140.45 mW. Lin et al. [19] proposed a new concept of intelligent mechanical energy harvesting consisting of gravity-driven rollers and a fishplate structure, using a magnetic coupling mechanism to boost the output power, ultimately enabling energy harvesting in an ultra-low-frequency environment of 0.1 Hz. Feng et al. [20] proposed a novel piezoelectric wave energy converter device that utilizes contactless technology, in which a spring pendulum provides a two-stage frequency amplification for low-frequency wave environments. Wang et al. [21] proposed a frequency up-conversion piezoelectric–electromagnetic hybrid wave energy harvester based on magnetic coupling, in which the excitation frequency can be increased by multiple rotating magnets. Under optimal conditions, the maximum power was 20.31 mW. Xia et al. [22] proposed a self-powered and self-sensing three-stage rotor electromagnetic generator wave energy harvester. The maximum average power output achieved was 8.24 W within a frequency range of 1 Hz. Liu et al. [23] proposed a frequency-increasing piezoelectric wave energy harvester (FPWEH) based on a gear mechanism and magnetic rotor. The FPWEH could generate an output voltage of 69.82 V and a maximum power of 28.33 mW for an external resistance of 20 k Omega.

Amplifying the low-frequency vibrations is a very effective way to increase power generation [24]. Magnetic coupling excitation has been widely used to amplify low-frequency vibrations, because when the vibration frequency of piezoelectric sheets is increased through magnetic coupling excitation, there is no direct contact between the magnet and the piezoelectric sheet, so there will be no friction loss involved. At the same time, this excitation method has the advantages of simple structure and flexible design, and efficient frequency amplification, so it has become popular recently [17,18,24,25,26,27,28,29]. The vibration of piezoelectric elements driven by magnetic excitation is a necessary condition for the device to produce electrical energy. A well-designed magnetic excitation component can significantly improve the power generation efficiency of the piezoelectric device. Therefore, it is important to study the influence of excitation magnets’ configuration and numbers and associated magnetic field conditions on the piezoelectric plate vibration.

## 2. Coupled Spring Pendulum and Piezoelectric Wave Energy Harvester

**Figure 1:**
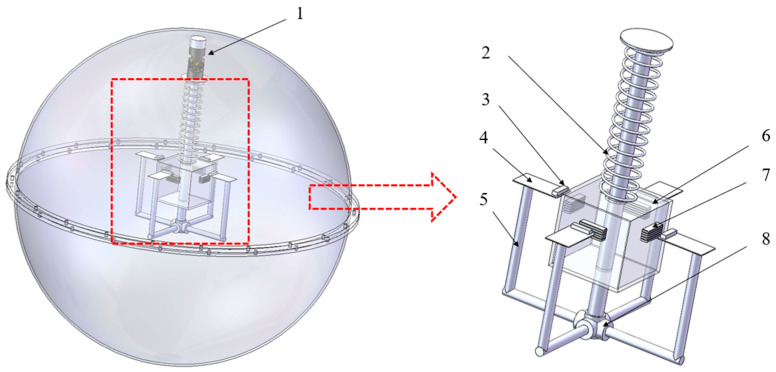
Layout of the coupled spring pendulum and wave energy piezoelectric harvester. 1—universal joint; 2—spring; 3—load magnet; 4—PZT-5H piezoelectric plate; 5—carbon fiber support rod; 6—square magnet carrier box; 7—excitation magnet; 8—five-way base.

The present piezoelectric energy harvester was designed to solve the power supply problem of ocean monitoring sensors. The power demand of most of these sensors is very low, so a wave energy device with a stable power of a few milliwatts provides sufficient energy supply, as shown in Figure 1.

The device comprises two modules, namely, the magnetic excitation module and the spring pendulum module [24,30]. The spring pendulum module consists of a universal joint (1), spring (2), square magnet carrier box (6), and excitation magnet (7). The magnetic excitation module is also a power generation module, which consists of a PZT-5H piezoelectric plate (4), carbon fiber support rod (5), five-way base (8), and load magnet (3) on the piezoelectric sheet. The magnetic poles of the magnet (3) are arranged opposite to those of the excitation magnet (7). 

Compared with existing devices, the coupled pendulum power generation device based on the piezoelectric effect proposed in this paper has the following advantages: First, the universal joint in the device enable the device to accept the wave force in all directions and absorb the wave energy efficiently. Second, the device has the secondary frequency amplification function of wave → spring pendulum → piezoelectric plate, which can effectively collect the low-frequency wave energy in the ocean relative to the resonance frequency. Finally, the non-contact magnetic excitation minimizes fatigue and ensures that the piezoelectric sheet has a long lifespan, while the small and simple structure configuration minimizes energy loss and manufacturing costs.

When the device encounters waves and it moves up and down and back and forth under the wave action, the spring pendulum module then starts swinging, resulting in up and down vibrations of the slider block. As a results, the excitation magnet vibrates up and down together with the slider in the spring pendulum, which in turn drives the load magnet fixed on the piezoelectric plate to move up and down at the end of the piezoelectric plate, and then the piezoelectric plate would vibrate to output electrical energy. The coupled spring pendulum and wave energy piezoelectric harvester is contained in a spherical shell, which can be driven by the wave forces in all directions in the ocean and floats well. The sphere is divided into two parts: the upper part is the carrier of the spring pendulum, and the lower part is mainly used to connect with the upper part, with sealant applied at the connection. The impact of the marine environment on the performance of this device is relatively small. The normal operating temperature range of piezoelectric elements is between −55 °C and +125 °C. The temperature change in the marine environment has little effect on the working state of the piezoelectric components. The normal working humidity range of piezoelectric components is usually 10% to 90% relative humidity. Taking Zhoushan sea area in China as an example, its annual average relative humidity is 80%, which has little effect on the piezoelectric components’ performance. The power generation system of the device is contained in a waterproof shell. Sealant or coating is used to protect the surface of the piezoelectric components from the marine and salt spray environment. The wiring is designed to avoid interfering with the movement of the internal pendulum and the vibration of the piezoelectric components.

Magnetic coupling excitation is critical for the performance of the device. It was found previously that each impact of the magnet causes the piezoelectric sheet to vibrate, and sometimes the vibration of piezoelectric sheets may become forced due to an excessively strong magnetic field, or the vibration may become weak due to an excessively weak magnetic field. At this time, the vibration frequency of the piezoelectric sheet would change, resulting in a decrease in power generation efficiency. To generate maximum electric output, the piezoelectric plate should approach resonance state under the action of magnetic excitation. The distribution of the magnetic field is critical to the effectiveness of magnetic excitation and, therefore, power generation. For example, the spacing, number, mass, and distribution of magnets can all affect the resulting magnetic field, and thereby the vibration of piezoelectric sheets. The objective of the present study was to conduct a magnetic excitation analysis experiment to identify the optimized magnetic excitation design for the coupled spring pendulum and wave energy piezoelectric harvester, which provides a self-powered system for ocean monitoring buoys. This system would eliminate the labor, time and cost of replacing batteries in ocean monitoring buoys while reducing environmental pollution.

## 3. Experimental Design and Principle

Magnetic excitation analysis experiments were conducted to examine the influence of magnet mass, spacing, quantity, and configuration on the power output of the piezoelectric plates.

### 3.1. Experimental Principle

**Figure 2:**
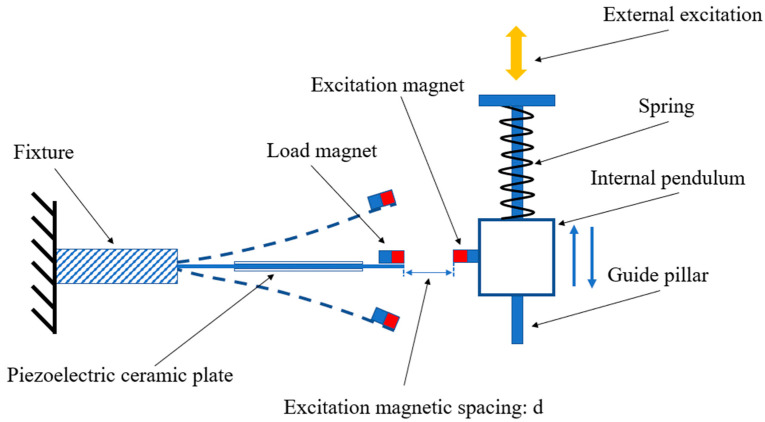
Schematic diagram of the magnetic excitation component.

Under the wave action, the internal pendulum in the magnetic excitation component underwent vertical motion relative to the piezoelectric plate under the action of the spring. Therefore, it was possible to simulate the power generation during the pendulum swinging process by using a magnet that moves up and down to impact the piezoelectric ceramic plate with a load magnet fixed at the end of the piezoelectric plate, as shown in Figure 2. We fixed one end of the piezoelectric plate and placed a load magnet on the other end to receive magnetic excitation and cause the piezoelectric plate to vibrate. External excitation was used to simulate the wave action on the device, which drove the inertia pendulum to swing and thus the spring to vibrate up and down. The guide column ensured the up and down reciprocating motion of the spring and inner pendulum. The excitation magnet and the load magnet with the same magnetic pole were placed opposite each other. Their horizontal distance represented the excitation magnetic spacing, and was recorded as d. When the excitation magnet moved up and down, the mutual repulsion of the same magnetic poles drove the load magnet continuously into motion, resulting in vibration and deformation of the piezoelectric ceramic sheet, and then continuously output electrical energy.

**Figure 3:**
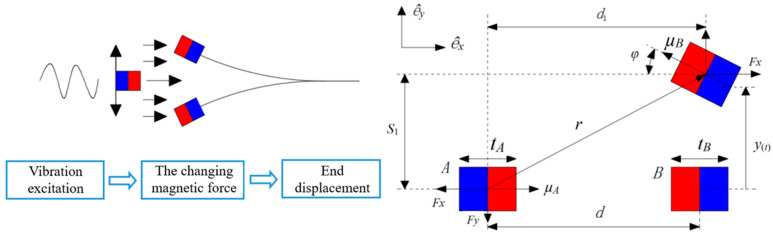
Diagram of the magnetic coupling nonlinear vibration system.

In the magnetic excitation component, the excitation magnet and the loaded piezoelectric ceramic plate can be regarded as a magnetic coupled nonlinear vibration system [31], as shown in Figure 3.

When the cantilever beam vibrated, the end permanent magnet moved a small distance *y* and a small rotation angle *φ*, which can be approximately expressed as $\varphi =\arcsin\left[w^{\prime }(L,t)\right]$. The horizontal distance between magnet *A* and magnet *B* is *d*_1_, and the vertical distance is *S*_1_:

(1) $$d_{1}=d+\frac{t_{A}}{2}-\frac{t_{A}\cos\varphi }{2}+L+\frac{t_{A}}{2}-\sqrt{{\left(L+\frac{t_{A}}{2}\right)}^{2}-y^{2}}$$

(2) $$s_{1}=y+\frac{t_{A}\sin\varphi }{2},$$

where *t_A_* is the thickness of permanent magnet *A*, and *d* is the horizontal distance of the permanent magnet. For the moving permanent magnet *A*, its magnetic moment vector is:

(3) $$\mu_{A}=-M_{A}V_{A}{\hat{e}}_{x}\cos\varphi +M_{A}V_{A}{\hat{e}}_{y}\sin\varphi ,$$

where *M_A_* is the magnitude of the magnetization vector of permanent magnet *A*, which can be calculated by the magnetic induction intensity *B_r_* of the permanent magnet, $M_{A}=B_{r}/\mu_{0}$, $\mu_{0}$ is the magnetic permeability of free space, $\mu_{0}=1.256\times 10^{-6}\;H/m$, and *V_A_* is the volume of permanent magnet *A*. For permanent magnet *B*, when y=0, its magnetic pole was opposite to that of permanent magnet *A*, and the magnetic moment vector was $\mu_{B}$:

(4) $$\mu_{B}=M_{B}V_{B}{\hat{e}}_{x},$$

where *M_B_* is the magnitude of the magnetization vector of permanent magnet *B*, which can be calculated by the magnetic induction intensity *B_r_* of the permanent magnet, $M_{B}=B_{r}/\mu_{0}$, and *V_B_* is the volume of permanent magnet *B*. Assuming the distance from $\mu_{B}$ to $\mu_{A}$ is given by:

(5) $$r=d_{i}{\hat{e}}_{x}+s_{i}{\hat{e}}_{y},$$

the magnetic field generated by permanent magnet *A* at the position of permanent magnet *B* can be expressed as:

(6) $$B=-\frac{\mu_{0}}{4\pi }\nabla \frac{\mu_{A}\cdot r}{\|r{\|}_{2}^{3}},$$

where ${\left\|\cdot \right\|}_{2}$ and ∇, respectively, represent the Euclidean norm and vector gradient operator [32]. The potential energy in a magnetic field can be expressed as:

(7) $$U_{m}=-B\cdot \mu_{B}\;.$$

By differentiating Equations (3)–(7) relative to r, the magnetic force can be obtained as:

(8) $$\begin{array}{l} F=-\nabla U_{m}=3M_{A}V_{A}M_{B}V_{B}\mu_{0}\cdot \\ \frac{\left[\left({\hat{\mu }}_{A}\cdot {\hat{\mu }}_{B}\right)\hat{r}+\left({\hat{\mu }}_{B}\cdot \hat{r}\right){\hat{\mu }}_{A}+\left({\hat{\mu }}_{A}\cdot \hat{r}\right){\hat{\mu }}_{B}-5\left({\hat{\mu }}_{A}\cdot \hat{r}\right)\left({\hat{\mu }}_{B}\cdot \hat{r}\right)\hat{r}\right]}{4\pi \|r{\|}_{2}^{4}} \end{array},$$

where ${\hat{\mu }}_{A}$, ${\hat{\mu }}_{B}$, and $\hat{r}$ are the vector units of $\mu_{A}$, $\mu_{B}$, and r, respectively. According to Equation (8), the components of magnetic repulsion in the *x* and *y* directions are given by:

(9) $$\begin{array}{l} F_{x}=\frac{3M_{A}V_{A}M_{s}V_{A}\mu_{0}}{4\pi {\left\{{\left[d+a(1-\cos\varphi )\right]}^{2}+{\left(y+a\sin\varphi \right)}^{2}\right\}}^{5/2}}\\ \cdot \{3[d+a(1-\cos\varphi )]\cos\varphi -(y+a\sin\varphi )\sin\varphi \\ +\frac{5\{-[d+a(1-\cos\varphi )]\cos\varphi +(y+a\sin\varphi )\sin\varphi \}{\left[d+a(1-\cos\varphi )\right]}^{2}}{{\left[d+a(1-\cos\varphi )\right]}^{2}+{\left(y+a\sin\varphi \right)}^{2}}\} \end{array}$$

(10) $$\begin{array}{l} F_{y}=\frac{3M_{A}V_{A}M_{B}V_{B}\mu_{0}}{4\pi {\left\{{\left[d+a(1-\cos\varphi )\right]}^{2}+{\left(y+a\sin\varphi \right)}^{2}\right\}}^{5/2}}\\ \cdot \{[d+a(1-\cos\varphi )]\sin\varphi -(y+a\sin\varphi )\cos\varphi -(y+a\sin\varphi )\sin\varphi \\ -\frac{5\{-[d+a(1-\cos\varphi )]\cos\varphi +(y+a\sin\varphi )\sin\varphi \}[d+a(1-\cos\varphi )][y+a\sin\varphi ]}{{\left[d+a(1-\cos\varphi )\right]}^{2}+{\left(y+a\sin\varphi \right)}^{2}}\}. \end{array}$$

From Equations (9) and (10), it can be seen that the magnetic force between magnets was complicated by the influence of magnet spacing and magnet volume. This also shows that it is very necessary to study how the configuration of magnet affects the magnetic excitation.

### 3.2. Experiment Setup

In the magnetic excitation system, the load magnet mass on the piezoelectric ceramic plate modulated the vibration frequency of the plate. The distance between the excitation magnet and load magnet, as well as the number and distribution of excitation magnets, determined the magnetic field, and they are all important factors affecting the power generation effect. Therefore, magnetic excitation experiments focus on these controlling factors for power generation.

**Figure 4:**
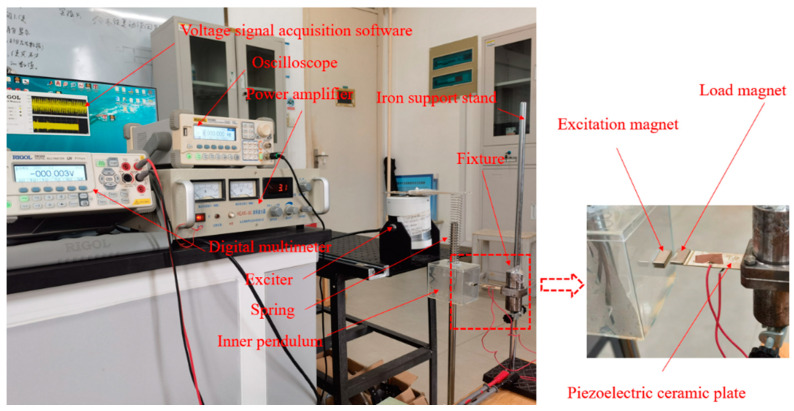
Magnetic excitation experimental system, consisting of an external excitation system, magnetic excitation power generation system, and voltage signal acquisition system.

The experiment was conducted in the laboratory, and the experiment setup is illustrated in Figure 4. The experiment consisted mainly of a HEAS-50 power amplifier (Nanjing Foneng Technology Industry Co., Ltd., Nanjing, China), DG1022U RIGOL oscilloscope (Puyuan Jingdian Technology Co., Ltd., Suzhou, China), HEV-50 exciter (Institute of vibration engineering, Nanjing Aeronautical University, Nanjing, China), DM3068 RIGOL digital multimeter (Puyuan Jingdian Technology Co., Ltd., Suzhou, China), iron frame, fixing device, piezoelectric ceramic plate with substrate, permanent magnet, internal pendulum, spring, wire, and guide column. Among them, the DM3068 RIGOL digital multimeter used in this experiment was designed for high precision, multi-function, and automatic measurements. It can collect data and display the trend chart of real-time measurement data. In the test, the time series of the output voltage signal waveform by the device was displayed in real time. The purpose of this experiment was to identify the mechanisms and influencing factors of power generation in the magnetic excitation system. Considering the lightweight design of the overall internal pendulum and the need for elucidating experimental phenomena, the parameter selection of the experiment is shown in the Table 1. Among them, a suitable magnetic excitation is necessary. Too large or too small excitation magnet will have a negative impact on the vibration of the piezoelectric plate. An excessive magnet size causes forced instead of free vibration of the piezoelectric plate and, therefore, mechanical fatigue and a shorter service life. When the magnet size is too small, however, the piezoelectric plate cannot produce a substantial resonance phenomenon. After many experiments, the optimum size for the excitation magnet was identified, which can provide sufficient excitation for the piezoelectric plate to produce a resonance phenomenon without causing forced vibration of the piezoelectric plate at the same time. The standardization of the magnet size was also a consideration. In the follow-up study, the magnet size will be customized according to the actual research needs.

The overall experimental investigation comprised three parts: (1) load magnet mass experiments, (2) excitation magnet spacing experiments, and (3) excitation magnet distribution experiments.

## 4. Experiment Results and Discussion

### 4.1. Load Magnets on Piezoelectric Ceramic Plates

In the magnetic excitation system shown in Figure 4, if the vibration frequency of the piezoelectric plate under magnetic excitation was close to the natural frequency, resonance would occur, the displacement amplitude of the piezoelectric plate would significantly amplify, and the power generation efficiency would be at its peak.

To produce resonance in piezoelectric ceramic plate movement, firstly, it was necessary to measure the natural vibration frequency of the piezoelectric ceramic plate under various loads. Subsequently, under the action of fixed magnetic excitation, the vibration frequency of the piezoelectric plate was measured and compared with the corresponding natural vibration frequency to identify the mass of the load magnet for resonance in the piezoelectric plate.

#### 4.1.1. Natural Vibration Frequency of Piezoelectric Plates

To measure the natural vibration frequency of the piezoelectric ceramic plate used, it was necessary to allow it to undergo free vibration. A small mass block was suspended from a piezoelectric ceramic plate by a lightweight thin rope to initiate the displacement of the piezoelectric plate. Then, the thin rope was cut to allow the mass block to fall freely from rest. Natural vibration frequency is the inherent property of the piezoelectric plate, which is independent of the magnitude of external excitation. As long as the piezoelectric plate produces free vibration under the gravity of the mass block, its natural frequency can be measured. At this point, the piezoelectric ceramic plate began to vibrate freely, and a digital multimeter was used to collect the voltage signal generated during this process.

**Figure 5:**
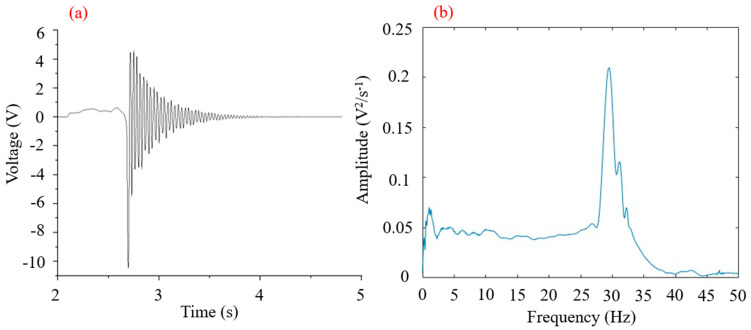
(**a**) Power generation during free vibration of piezoelectric ceramic plates. (**b**) Spectrum of piezoelectric ceramic plate power generation during free vibration.

In many previous experiments of natural frequency of piezoelectric plates, the results of natural frequency were consistent with each other. A free vibration experiment with only the piezoelectric ceramic plate itself under no load was conducted, and the test results are shown in Figure 5.

From Figure 5, it can be seen that the power generation of the piezoelectric ceramic sheet displayed a trend of oscillation decay during free vibration due to the attenuation in the free vibration. The amplitude in Figure 5b shows the result of the accumulation of various frequencies when the piezoelectric ceramic plate was vibrating. The frequency spectrum in Figure 5 shows that the natural vibration frequency of the piezoelectric plate was around 29 Hz.

**Figure 6:**
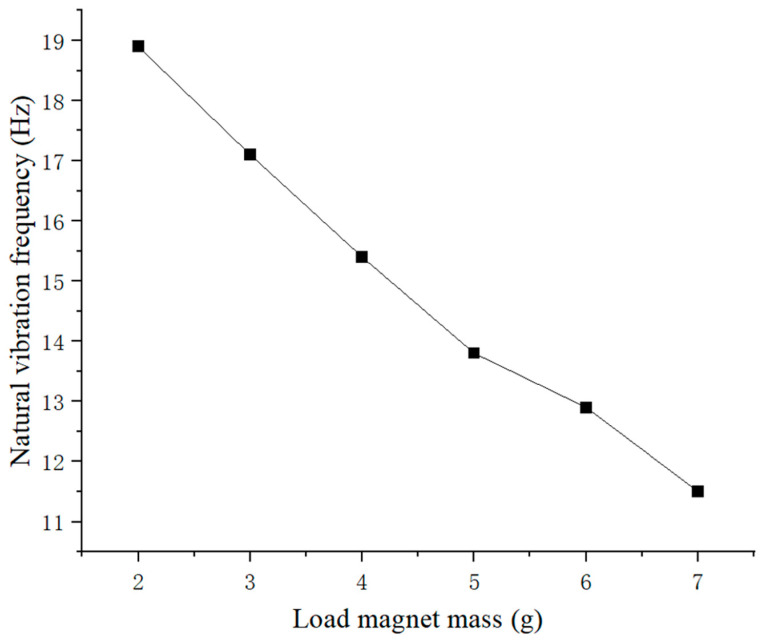
Natural vibration frequency of piezoelectric ceramic plates vs. load magnet mass.

The same free vibration experiment was repeated for load magnets of different masses between 2 g and 7 g on piezoelectric ceramic plates, while keeping other conditions unchanged. Subsequently, Fourier transform was performed on the voltage signals collected from each experiment, and the results are shown in Figure 6.

The relationship between the natural vibration frequency of piezoelectric plates and load magnet mass is shown in Figure 6. It is evident from Figure 6 that compared to the unloaded case, once a 2 g loaded magnet was placed on the piezoelectric ceramic plate, the natural vibration frequency of the piezoelectric plate was reduced by about one-third to around 19 Hz. As the load magnet mass on the piezoelectric plate gradually increased from 2 g to 7 g, the natural vibration frequency gradually decreased, from around 19 Hz to below 12 Hz. Based on this result, it can be inferred that the natural frequency of the piezoelectric plate as a whole would continue to decrease with the loaded magnet mass. Therefore, it is advisable to choose a lighter-weight magnet as much as possible in order to achieve resonance as a whole.

#### 4.1.2. Vibration Frequency of Piezoelectric Plates Under Magnetic Excitation

After obtaining the natural free vibration frequencies of piezoelectric plates under different loads, the vibration frequencies of piezoelectric plates under magnetic excitation were derived to determine the load magnet mass that can achieve the resonance conditions.

**Figure 7:**
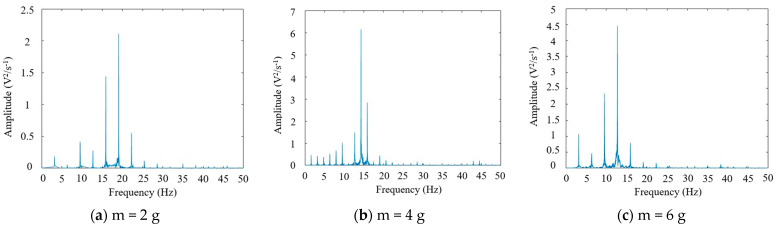
Frequency spectra of piezoelectric ceramic plates with different load magnet masses under magnetic excitation. (**a**) For a loaded magnet mass of 2 g, the main vibration frequency of the piezoelectric ceramic plate was about 19 Hz. (**b**) For a magnet mass was 4 g, the main vibration frequency of the piezoelectric ceramic plate was about 14 Hz. (**c**) For a loaded magnet mass of 6 g, the main vibration frequency of the piezoelectric ceramic plate was about 12 Hz.

**Figure 8:**
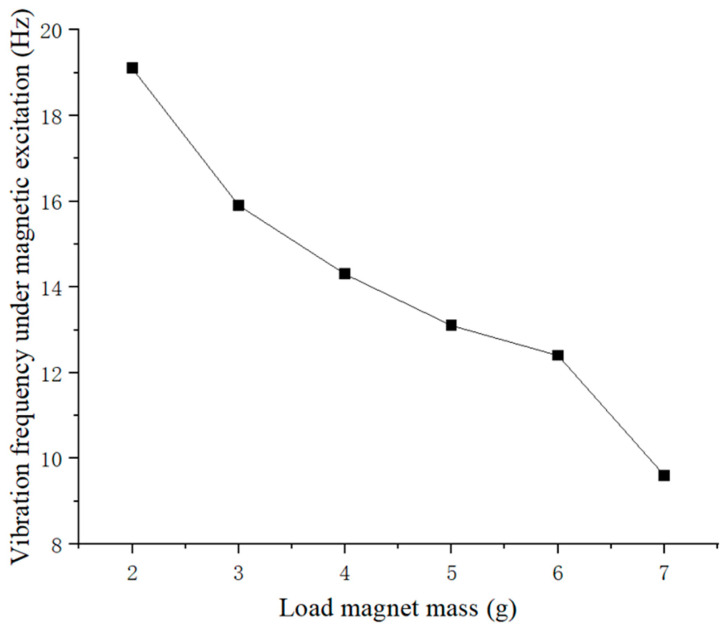
Vibration frequencies of piezoelectric ceramic plates vs. load magnet mass under magnetic excitation.

When using an excitation magnet as a fixed external magnetic excitation, vibration experiments were conducted on piezoelectric ceramic plates under different loads again. Subsequently, Fourier transform was performed on the voltage signals collected from each experiment, and the results are shown in Figure 7 and Figure 8.

**Figure 9:**
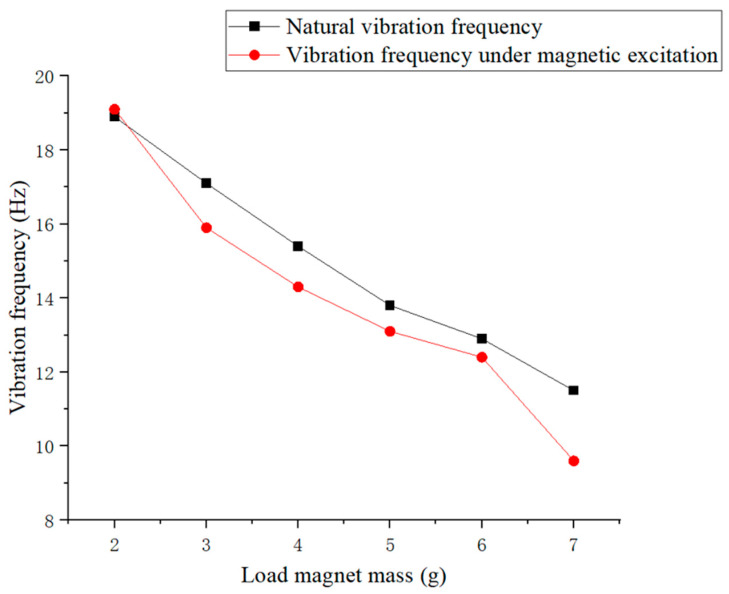
Comparison of the relationship between the natural frequency of the piezoelectric plate (black) and the vibration frequency of the piezoelectric plate with external magnetic excitation (red) under different load magnet masses.

Figure 7 shows that under the coupling of magnetic excitation and self-vibration, the vibration of the piezoelectric ceramic sheet exhibited multiple frequency peaks. From Figure 8, for a fixed external magnetic excitation, as the mass of the loaded magnet on the piezoelectric ceramic sheet gradually increased from 2 g to 7 g, the overall vibration frequency still gradually decreased from 19 Hz to around 9 Hz. When the mass of the loaded magnet exceeded 6 g, the overall vibration frequency of the piezoelectric plate suddenly decreased. Under the same magnetic excitation conditions, it was more difficult for a heavier load magnet to generate the optimum power generation of the piezoelectric plate. Subsequently, the natural vibration frequency of the piezoelectric ceramic plate corresponding to the load magnet mass ranging from 2 g to 7 g was compared with its vibration frequency under magnetic excitation, as shown in Figure 9.

From Figure 9, it can be seen that when the load magnet mass was 2 g, under the action of the external excitation magnet, the vibration frequency of the piezoelectric plate was also around 19 Hz, which is almost identical to its natural vibration frequency without external magnetic excitation. It is evident that the piezoelectric plate reached a resonant state at this time, with peak power generation performance. When the load magnet mass was 3 g, 4 g, 5 g, 6 g, and 7 g, under the same magnetic excitation, the vibration frequency of the piezoelectric ceramic piece could not reach its corresponding natural vibration frequency to achieve resonance. Among them, the difference between the two vibration frequencies was the largest when the mass of the loaded magnet was 7 g. It can be concluded that under the current experimental conditions, only when the mass of the loaded magnet was 2 g did the piezoelectric ceramic sheet exhibit resonance under the specific external magnetic excitation. So, the load magnet mass of 2 g will be used in the subsequent experiments.

### 4.2. Distance Between Excitation and Load Magnet

In the previous experiment, the load magnet mass that could induce resonance in the piezoelectric plate displacement was determined, which is the prerequisite for the optimal power generation of the device. However, the distance between the excitation and the load magnet is also an important factor for the power generation of the piezoelectric plate. The reason is that the excitation magnet layout affects the magnetic field and, therefore, the magnetic force between the magnets and the vibration of the piezoelectric plates, and thus their power generation. Therefore, the optimal magnetic excitation layout should be determined through experiments to enable piezoelectric plates to achieve the best power generation in resonance.

**Figure 10:**
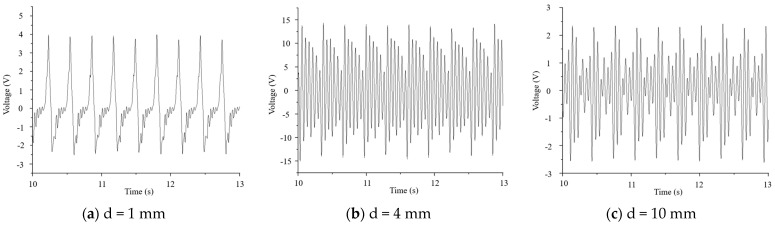
Voltage signals of piezoelectric ceramic plates under different distances between excitation and load magnets. (**a**) At an excitation magnet spacing of 1 mm, the piezoelectric plate showed the phenomenon of forced vibration. (**b**) At an excitation magnet spacing of 4 mm, the piezoelectric plate showed the phenomenon of vibration attenuation, which was close to free vibration. (**c**) At an excitation magnet spacing of 10 mm, the vibration attenuation of the piezoelectric plate became weaker.

In the case of a load magnet mass of 2 g, if there was only one excitation magnet, the distance between the excitation and the load magnet gradually increased from 1 mm to 10 mm, and the voltage signals of the piezoelectric ceramic plate are shown in Figure 10.

**Figure 11:**
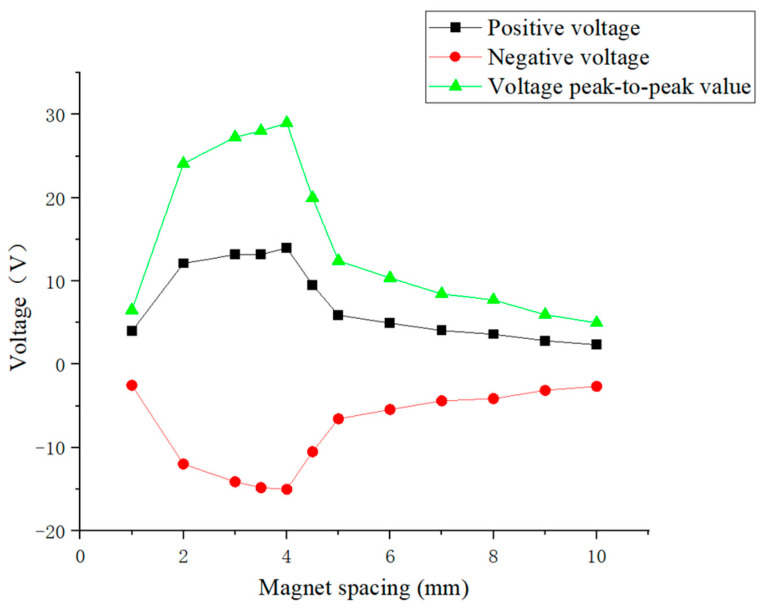
Maximum voltages of piezoelectric ceramic plates under different excitation magnet spacing.

Due to the presence of the piezoelectric effect, when piezoelectric elements are subjected to external forces, polarization phenomena will occur inside them. When the direction of force is consistent with the polarization direction of the piezoelectric element, a positive voltage will be generated, and when the direction of force is opposite to the polarization direction, a negative voltage will be generated. Due to the continuous up and down movement of the excitation magnet, the direction of the force on the load magnet was also changing, resulting in alternating positive and negative voltages from the piezoelectric plate. From Figure 11, it can be seen that when the excitation magnet spacing was 2 mm to 4 mm, the maximum voltage first slowly increased with the increase in spacing. Then, when the excitation magnet spacing was between 4 mm and 10 mm, the maximum voltage decreased with increasing spacing. When the distance between the excitation and load magnet was 1 mm, the piezoelectric ceramic plate generated the minimum voltage. When the distance between the excitation and load magnet was 4 mm, the piezoelectric ceramic plate generated the maximum voltage, with a peak-to-peak voltage of 28.89 V. When the distance between the excitation and load magnet was 1 mm, the larger magnetic force caused the piezoelectric ceramic plate to undergo forced vibration, manifested as a sudden voltage change, so the voltage generated was relatively weak. When the magnetic excitation spacing was 2 mm to 4 mm, the vibration of the piezoelectric ceramic plate was relatively close to its free vibration, showing obvious voltage oscillation attenuation, indicating that the resonance state of the piezoelectric ceramic plate was strong, and the voltage peak-to-peak values were relatively large at these times. Once the magnetic excitation spacing was 5 mm or more, the voltage signal of the piezoelectric ceramic piece underwent a sudden change from high to low, indicating that its resonance state became weaker and the voltage peak value decreased with increasing magnetic excitation spacing. Subsequently, frequency spectrum analysis of the voltage signal was conducted to explore the variation in the vibration frequency of the piezoelectric ceramic plate with the excitation magnetic spacing.

**Figure 12:**
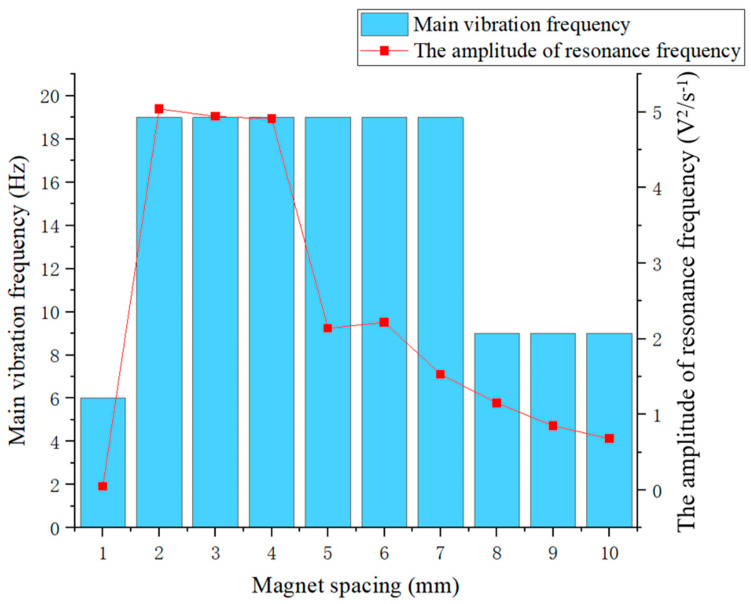
The main vibration frequency (blue) and voltage amplitude at resonance frequency (red) of piezoelectric ceramic plates under excitation magnet spacing of 1 to 10 mm.

Figure 12 shows that when the excitation magnet spacing was 1 mm, the main vibration frequency of the piezoelectric ceramic plate was 6 Hz, and no resonance phenomenon occurred because the piezoelectric ceramic plate was in a forced vibration state at this time. As the excitation magnet spacing gradually increased, the piezoelectric ceramic plate began to exhibit a resonance phenomenon, but the voltage amplitude at the resonance frequency showed an overall decreasing trend. When the excitation magnet spacing was between 2 mm and 4 mm, the voltage amplitude at the resonance frequency of the piezoelectric ceramic sheet was high, while the decrease with excitation magnet spacing was slow, indicating that the resonance state was good at this time. Once the magnetic excitation spacing was 5 mm or more, the coupling was weakened due to reduced magnetic force, the voltage amplitude at the resonance frequency of the piezoelectric ceramic plate decreased significantly, and the decay rate with increasing excitation magnet spacing accelerated. It can be seen that the main vibration frequency of the piezoelectric ceramic plate gradually changed to between 9 and 10 Hz, so the resonance state of the piezoelectric ceramic plate gradually weakened and disappeared. This phenomenon is correlated with the variation of the voltage generated by piezoelectric ceramic plates, indicating that the latter is mainly influenced by voltage amplitude at the resonance frequency.

**Figure 13:**
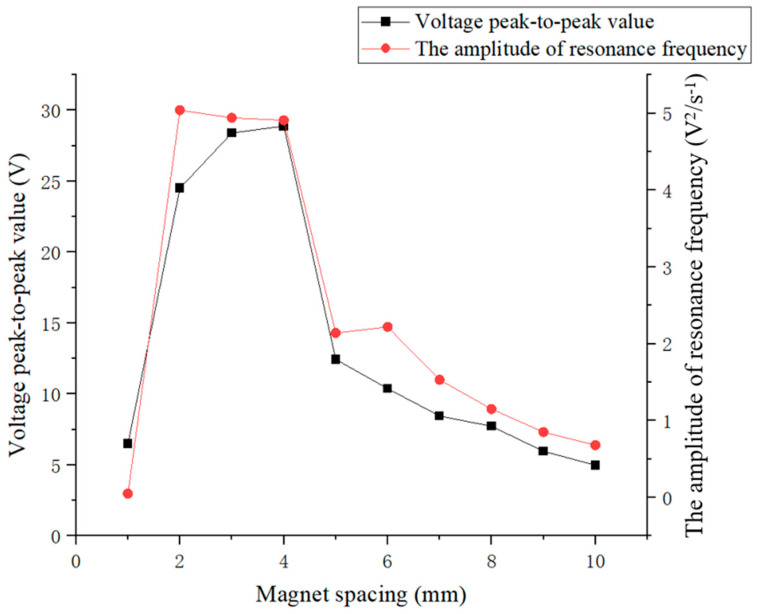
The relationship between the voltage amplitude at the resonance frequency of piezoelectric ceramic plates (red) and their peak-to-peak voltage (black) for magnet spacing of 1 to 10 mm.

Figure 13 illustrates the close correlation between the voltage generated by piezoelectric ceramic plates and the voltage spectrum amplitude at their resonance frequency: a higher voltage amplitude at the resonance frequency of the piezoelectric ceramic plate resulted in a higher voltage output. At the excitation magnet spacing of 1 mm, the piezoelectric ceramic plate did not resonate, and the voltage generated was also very low. When the excitation magnet spacing was between 5 mm and 10 mm, the amplitude at the resonance frequency of the piezoelectric ceramic plate and voltage output were both very low and continuously decreased with increasing spacing. However, at the excitation magnet spacing between 2 mm and 4 mm, the amplitude at the resonance frequency and the voltage output of the piezoelectric ceramic plate both attained the peak values. The former decreased slightly with the spacing, while the latter increased slightly with spacing. Among them, at the excitation magnet spacing of 4 mm, the piezoelectric ceramic plate generated the maximum voltage, and the voltage amplitude at the resonance frequency of the piezoelectric ceramic plate was also relatively high, indicating that the piezoelectric ceramic plate achieved the optimum power generation in the resonance state. Therefore, in the subsequent experiments, the excitation magnet spacing will be fixed at 4 mm.

### 4.3. Excitation Magnets Configuration

The aforementioned experiments determined the load magnet mass for resonance vibration in the piezoelectric plate and the excitation magnet spacing for maximum power generation at the resonance state. However, the number and spatial distribution of excitation magnets can also change the magnetic field between the excitation and load magnets, which determines the strength of the magnetic excitation and, therefore, the piezoelectric plate vibration, and ultimately the power generation. Next, the number and spatial distribution of excitation magnets will be identified for optimum magnetic excitation performance.

#### 4.3.1. Single-Row Magnets

**Figure 14:**
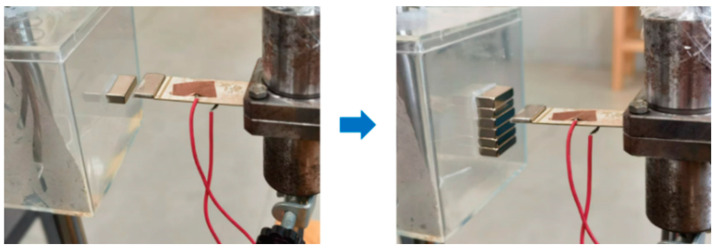
Excitation magnets arranged vertically in a single-row experiment.

The most common arrangement of magnets is several identical magnets pulled together by magnetic force, transforming from small magnets into a large magnet, as shown in Figure 14. As a result, the magnetic field will be enhanced, and the magnetic excitation and the piezoelectric ceramic sheet vibration will, in turn, change accordingly. Therefore, the response of power generation to the excitation magnet numbers and arrangement will be examined next.

**Figure 15:**
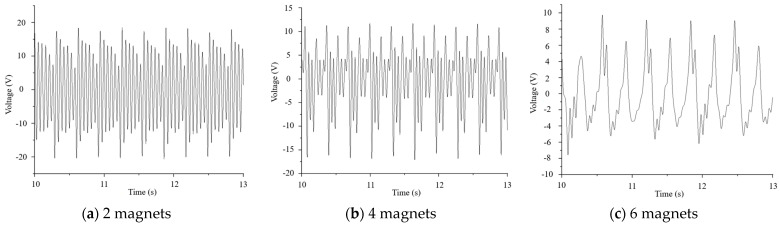
Voltage signals of piezoelectric ceramic plates with 1 to 6 excitation magnets stacked vertically in a row. (**a**) For 2 magnets, the piezoelectric plate showed the vibration attenuation, which was close to free vibration. (**b**) For 4 magnets, the vibration attenuation of the piezoelectric plate became weaker. (**c**) For 6 magnets, the vibration attenuation of the piezoelectric plate disappeared completely.

Under the conditions of a load magnet mass of 2 g and an excitation magnet spacing of 4 mm, the number of excitation magnets was gradually increased from 1 to 6 on the slider attached to the inner pendulum, and power generation induced by piezoelectric plate vibration is shown in Figure 15.

**Figure 16:**
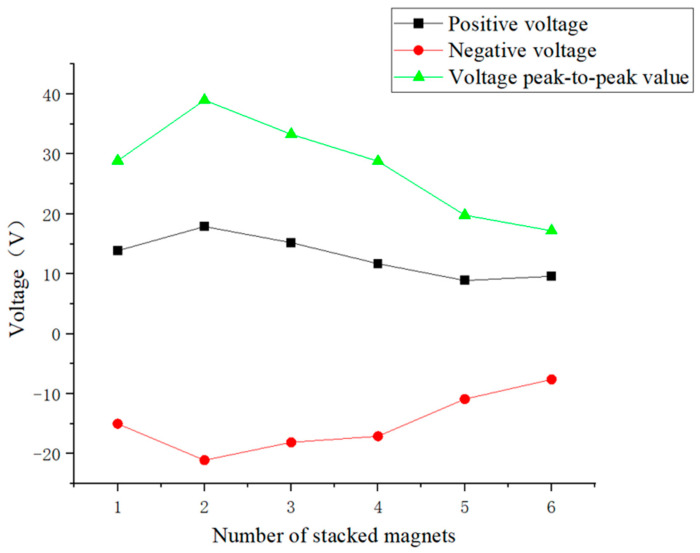
Maximum voltage of piezoelectric ceramic plates under 1 to 6 stacked magnets.

From Figure 16, it can be seen that as the number of stacked magnets increased, the generated voltage of the piezoelectric ceramic sheet first increased and then decreased. When the number of stacked magnets was 2, the generated voltage reached its maximum, and the corresponding peak-to-peak voltage was 39 V. When one, two, and three magnets were stacked, the voltage signal of the piezoelectric ceramic plate during vibration was almost the same as that during free vibration, showing obvious oscillation attenuation, indicating that the resonance state of the piezoelectric plate was relatively good at this time, so the voltage was relatively high. When four magnets were stacked, the oscillation of the voltage signal of the piezoelectric ceramic plate was weakened, so was the resonance phenomenon and, therefore, the voltage also decreased. When the number of stacked magnets increased to 5 and 6, the mass and magnetic field of the entire excitation magnet became too large. The excessive gravity and magnetic field of the excitation magnet directly constrained the vibration of the spring and suppressed the magnetic excitation. The piezoelectric plate could not reach the resonance state, so that the voltage at this time was smaller, and the voltage signal did not display the vibration attenuation pattern at all.

#### 4.3.2. Magnet Arrays

**Figure 17:**
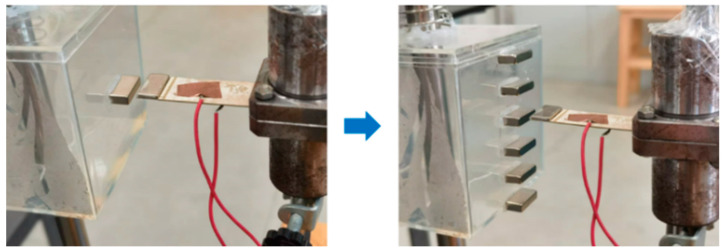
Excitation magnet array experiment.

A magnet array with several identical magnets arranged at the same spacing is common, as shown in Figure 17. Due to the magnetic induction intensity being a vector, magnetic fields generated by each magnet of magnet arrays will interteract with each other, and the magnetic excitation and the piezoelectric sheet vibration are different and more complex than those of a single magnet case. Therefore, it is necessary to examine the power generation response to magnet array excitations.

**Figure 18:**
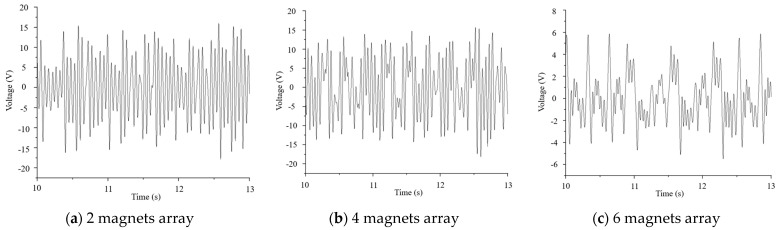
Voltage signals of piezoelectric ceramic plates under an array with 1 to 6 magnets. (**a**) When the number of magnets was 2, the piezoelectric plate showed the vibration attenuation. (**b**) When the number of magnets was 4, the vibration attenuation of the piezoelectric plate became weaker. (**c**) When the number of magnets was 6, the vibration attenuation of the piezoelectric plate disappeared completely.

Under the same conditions of a load magnet mass of 2 g and an excitation magnet spacing of 4 mm, the number of excitation magnets in the arrays was gradually increased from 1 to 6, and the time history of vibration power generation of piezoelectric ceramic plates is shown in Figure 18.

**Figure 19:**
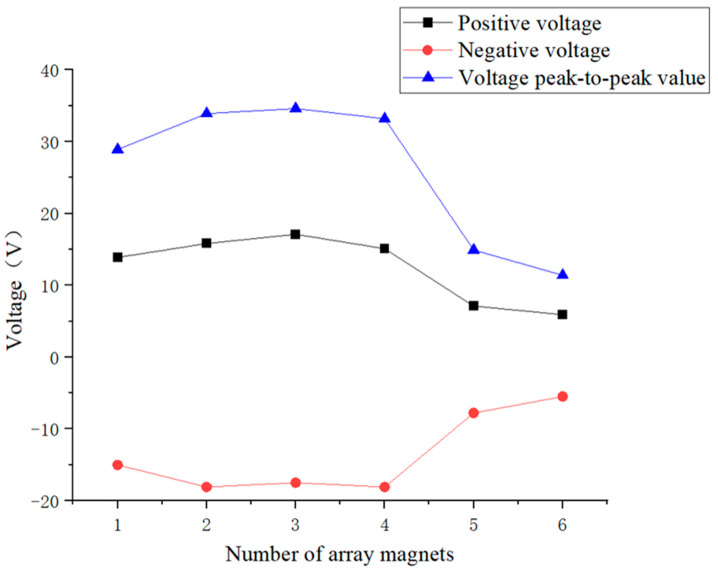
Maximum voltage of piezoelectric ceramic plates under an array with 1 to 6 magnets.

Figure 19 shows that as the number of array magnets increased, the voltage of the piezoelectric ceramic piece slowly increased first and then decreased. The voltage reached the maximum when the number of array magnets was 3, and the corresponding peak-to-peak voltage was 34.6 V. When the number of array magnets was 1, 2, and 3, the oscillation attenuation phenomenon of the piezoelectric ceramic plate voltage was significant, indicating that the resonance state of the piezoelectric ceramic plate was better and the voltage was higher. When the number of array magnets exceeded 3, the magnetic fields of multiple magnets interfered with each other, and the mass of the entire inner pendulum also increased, resulting in a decrease in the magnetic excitation. The vibration attenuation of the piezoelectric ceramic plate voltage gradually decreased to nearly zero, so the voltage decreased.

**Figure 20:**
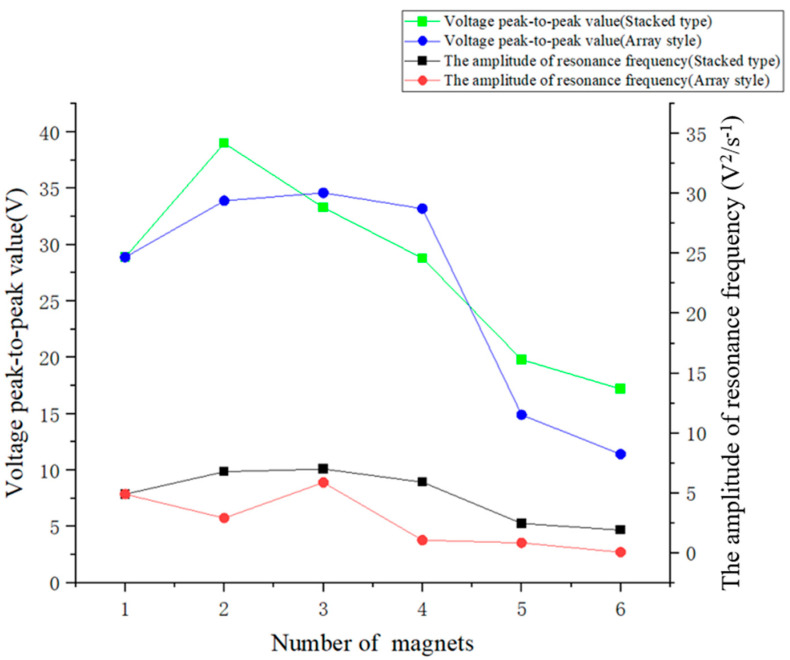
Comparison of peak-to-peak voltage and amplitude at resonance frequency of the piezoelectric ceramic plate under an array or stacked with 1 to 6 excitation magnets.

Figure 20 shows that regardless whether the excitation magnets were arranged in a row or array on the slider of the inner pendulum, when the number of magnets was more than 3, the maximum voltage amplitude at resonance frequency of the piezoelectric ceramic plate decreased. Therefore, the number of excitation magnets on the inner pendulum should not exceed 3. Secondly, regardless the number of excitation magnets, the voltage amplitude at resonance frequency of the piezoelectric ceramic plate was always higher when the excitation magnets were arranged in a row than in an array. Therefore, under the action of magnets in a vertical row, the piezoelectric ceramic plate could achieve better resonance state and optimal power generation performance. Finally, when two excitation magnets were arranged in a vertical row, the maximum peak-to-peak voltage generated by the piezoelectric ceramic sheet was 39 V. In summary, when two excitation magnets were stacked on top of each other vertically, the piezoelectric ceramic sheet exhibited the best power generation performance.

## 5. Conclusions

A set of comprehensive magnetic excitation experiments was carried out to investigate the influence of magnetic excitation configuration on the power generation of the piezoelectric plate in a contactless coupled spring pendulum and piezoelectric wave energy harvesting system. The overall experiment consisted of three parts: the load magnet mass on the piezoelectric ceramic sheet, the distance between the excitation and load magnet, and the number and spatial layout of the excitation magnet on the inner spring pendulum. The optimal loading magnet mass to induce the resonance phenomenon in piezoelectric ceramic plates was found to be 2 g. The 4 mm excitation magnet spacing enabled the piezoelectric ceramic sheet to have the best power generation performance in the resonance state. Two excitation magnets stacked vertically on the inner pendulum could further improve the power generation performance of the piezoelectric ceramic sheets, as shown in Table 2.

Finally, under the conditions of a load magnet mass of 2 g, excitation magnet spacing of 4 mm, and two excitation magnets stacked vertically on the inner pendulum, the power generation of the piezoelectric ceramic sheet attained the maximum, resulting in a peak-to-peak voltage of 39 V. Under the optimal configuration, when the piezoelectric plate resonates, it will produce elastic deformation and minimal mechanical fatigue. This will have an effect on the service life of the piezoelectric plate. After the whole device system is improved and established, a further durability study will be carried out. This study experimentally derived the optimal magnetic excitation configuration for the contactless coupled spring pendulum and piezoelectric wave energy harvester. This method provides new insight for magnetic excitation for other energy harvesters and increases novel techniques for converting wave energy into electrical energy more effectively. The ultimate purpose of the device proposed in this paper is to supply power for low-power equipment, such as marine monitoring sensors. The focus of the series of experiments in this paper was to determine the optimal structural parameters based on the power output voltage generated by the piezoelectric plate. The next step in future work will focus on load matching, the actual power output of the whole system, and the power generation performance of the device in the real marine environment. After continuous improvement of the whole system through series of experiments, it will perform as expected in low-power equipment, such as marine monitoring sensors.

## Figures and Tables

**Table 1:** Main experimental equipment parameters.

Experimental Equipment	Parameter
Exciter	Excitation frequency 3 Hz; amplitude ± 5 mm
Spring	Stiffness 35 N/m; length 14 cm
Internal pendulum	Size 80 × 80 × 80 mm; quality 78 g
Excitation magnet	Size 20 × 10 × 4 mm; quality 6 g
Piezoelectric ceramic sheet	Size 60 × 20 × 0.2 mm

**Table 2:** The optimal structural parameters.

Structure Parameters	State of Piezoelectric Element	Voltage
Load magnet mass: 2 g	Resonance	/
Excitation magnetic spacing: 4 mm	Resonance	28.89 V
Excitation magnet distribution: stacking 2 magnets	Resonance	39 V

## Data Availability

Data are contained within the article.
